# Ginsenoside Rb1 Treatment Attenuates Pulmonary Inflammatory Cytokine Release and Tissue Injury following Intestinal Ischemia Reperfusion Injury in Mice

**DOI:** 10.1155/2015/843721

**Published:** 2015-06-16

**Authors:** Ying Jiang, Zhen Zhou, Qing-tao Meng, Qian Sun, Wating Su, Shaoqing Lei, Zhengyuan Xia, Zhong-yuan Xia

**Affiliations:** ^1^Department of Anesthesiology, Renmin Hospital of Wuhan University, Wuhan 430060, China; ^2^Department of Cardiovascular Surgery, Renmin Hospital of Wuhan University, Wuhan 430060, China; ^3^Department of Anesthesiology, The University of Hong Kong, Pok Fu Lam, Hong Kong; ^4^Department of Anesthesiology, Affiliated Hospital of Guangdong Medical College, Zhanjiang, Guangdong 524001, China

## Abstract

*Objective*. Intestinal ischemia reperfusion (II/R) injury plays a critical role in remote organ dysfunction, such as lung injury, which is associated with nuclear factor erythroid 2-related factor 2 (Nrf2)/heme oxygenase-1 (HO-1) signaling pathway. In the present study, we tested whether ginsenoside Rb1 attenuated II/R induced lung injury by Nrf2/HO-1 pathway. *Methods*. II/R injury was induced in male C57BL/6J mice by 45 min of superior mesenteric artery (SMA) occlusion followed by 2 hours of reperfusion. Ginsenoside Rb1 was administrated prior to reperfusion with or without ATRA (all-transretinoic acid, the inhibitor of Nrf2/ARE signaling pathway) administration before II/R. *Results*. II/R induced lung histological injury, which is accompanied with increased levels of malondialdehyde (MDA), interleukin- (IL-) 6, and tumor necrosis factor- (TNF-) *α* but decreased levels of superoxide dismutase (SOD) and IL-10 in the lung tissues. Ginsenoside Rb1 reduced lung histological injury and the levels of TNF-*α* and MDA, as well as wet/dry weight ratio. Interestingly, the increased Nrf2 and HO-1 expression induced by II/R in the lung tissues was promoted by ginsenoside Rb1 treatment. All these changes could be inhibited or prevented by ATRA. *Conclusion*. Ginsenoside Rb1 is capable of ameliorating II/R induced lung injuries by activating Nrf2/HO-1 pathway.

## 1. Introduction

Intestinal ischemia reperfusion (II/R) injury is a life-threatening clinical surgical emergency, which is associated with the exacerbation of intestinal injury and a systemic inflammatory response leading to progressive distal organ impairment, finally resulting in cardiocirculatory, respiratory, hepatic, and renal failure. Acute respiratory distress syndrome (ARDS) induced by lung injury is one of the most serious complications. These clinical problems were involved in diverse causes such as intestinal barrier damage, bacteria translocation and oxidative stress, and activation of multiple inflammatory mediators [1, 2]. However, there still remain many doubts in the pathophysiology and therapeutics of II/R induced remote organ injury, especially lung injury.

Ginsenoside Rb1, a major active constituent of ginseng (*Panax ginseng*), has antioxidative effects and has been demonstrated to protect multiple organs from ischemia reperfusion injury [3–9]. However, it has not been fully elucidated whether it can also attenuate II/R induced acute lung injury. Nuclear factor erythroid 2-related factor 2 (Nrf2)/antioxidant response element (ARE) signaling pathway has been found as the most important endogenous antioxidative stress mechanism. It has been reported that Nrf2/ARE signaling pathway performs a fundamental role in protecting the body against the xenobiotics and oxidative injury in the pathophysiology of digestive system, circulation system, nervous system, and immune system diseases [10–13]. Nrf2 is a nuclear transcription factor that controls the expression and coordinates induction of a battery of defensive genes encoding detoxifying enzymes and antioxidant proteins [14]. In response to stimulation of oxidative stress, Nrf2 translocates from the cytoplasm into the nucleus and then binds to a* cis*-acting enhancer sequence designated as ARE and regulates ARE mediated antioxidant enzyme gene such as heme oxygenase-1 (HO-1) expression and induction [15, 16]. HO-1 belongs to a member of the heat shock protein family and plays a significant protective role against inflammatory processes and oxidative tissue injury [17].

In this study, we established a superior mesenteric artery (SMA) occlusion/reperfusion mice model to induce lung injury. We used ATRA (all-transretinoic acid) as inhibitor of Nrf2/ARE signaling pathway, which interfered in the recruitment of Nrf2 to the ARE, thus disrupting the activation of ARE-driven genes [18]. With the treatment of ginsenoside Rb1, we aim to investigate whether ginsenoside Rb1 attenuates acute lung injury (ALI) induced by II/R in mice via Nrf2/ARE pathway.

## 2. Material and Methods

### 2.1. Mice

The current study was approved by the Animal Care Committee of Wuhan University, China, and was performed in accordance with National Institutes of Health guidelines for the use of experimental animals. Male C57BL/6 mice (9–12 weeks old; 17–22 g) were purchased from HUNAN SLAC JD Laboratory Animal Co. Ltd., China. They were housed under standard laboratory conditions at 22–24°C, relative humidity of 50 ± 15%, and kept on a 12 h day/night rhythm with free access to water and food. All experimental protocols conducted in the mice were carried out in accordance with the Guide for the Care and Use of Laboratory Animals by the National Institutes of Health (NIH Publication number 80-23).

### 2.2. Surgical Preparation

Animals were anesthetized intraperitoneally with pentobarbital sodium (50 mg/kg body weight). A midline laparotomy was performed; then the superior mesenteric artery (SMA) was isolated. The II/R injury was established by occluding SMA with a microvascular clip for 45 minutes followed by 2 hours of reperfusion as previously described [19]. Ischemia was recognized by the existence of pulseless or pale color of the small intestine. The return of pulses and the reestablishment of the pink color were assumed to indicate valid reperfusion of the intestine. The Sham group underwent the same surgical process, apart from occlusion of SMA. After 2 h reperfusion, the mice were killed. A median sternotomy was performed; the lung and intestine samples were obtained for further analysis.

### 2.3. Experimental Protocol

**Figure 1:**
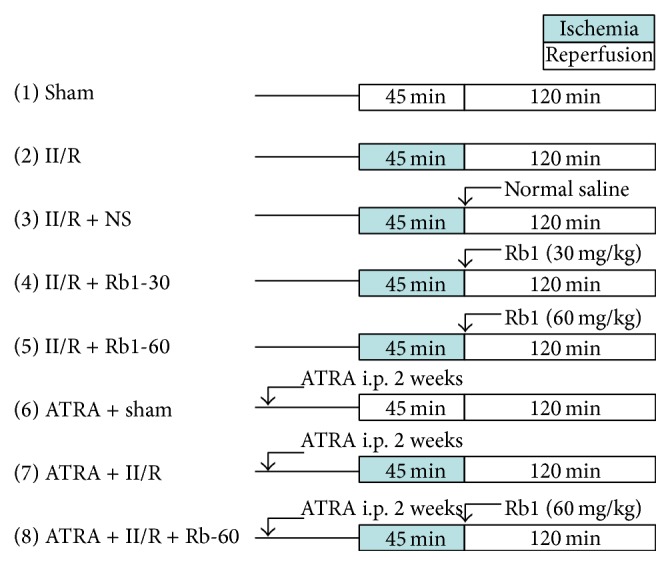
Experimental protocols. Mice were subjected to 45 min of SMA occlusion followed by 2 h of reperfusion. II/R: intestinal ischemia/reperfusion, NS: normal saline, Rb1: ginsenoside Rb1, and ATRA: all-transretinoic acid.

The mice were randomly allocated into eight groups (*n* = 8 in each group) (Figure 1): (1) Sham surgical preparation including isolation of the SMA without occlusion was performed (Sham); (2) mice were subjected to II/R without treatment (II/R); (3) mice were subjected to II/R with treatment of normal saline 10 minutes before reperfusion (II/R + NS); (4), (5) mice were treated with 30 mg/kg (II/R + Rb1-30) or 60 mg/kg (II/R + Rb1-60) ginsenoside Rb1, in which surgery was performed as in the II/R group with administration of the ginsenoside Rb1 intraperitoneally 10 minutes before reperfusion; (6) mice were subjected to Sham surgery and treated with ATRA (ATRA + Sham), which is the inhibitor of Nrf2/ARE signaling pathway; (7) mice were subjected to II/R and treated with ATRA (ATRA + II/R); (8) mice were subjected to II/R and treated with ATRA and 60 mg/kg ginsenoside Rb1 as group 5 (ATRA + II/R + Rb1-60). During the last two weeks before the operation, the mice in the group 6, 7, 8 received ATRA i.p. daily at 10 mg/kg and fed on a vitamin A-deficient diet, and the mice in the other groups received the equivalent volume of corn oil and fed on a control normal diet [18].

### 2.4. Lung Histology

The left lung was removed and fixed in 10% formalin. Following embedding in paraffin, the sections of 4 *μ*m were stained with hematoxylin and eosin for light microscopy. Semiquantitative analysis of lung histopathology was performed by scoring the tissues based on lung edema, infiltration of inflammatory cells, alveolar hemorrhage, hyaline membrane, and atelectasis: no lesion, 0; injured area ⩽ 25%, 1; injured area 26–50%, 2; injured area 51–70%, 3; injured area 71–90%, 4; injured area > 90%, 5. A total of three fields were randomly selected for each slide and the average was used as the histopathology score [20].

### 2.5. Histopathological Assessment of Intestines

After reperfusion, 1 cm of small intestine without adipose tissue was taken from the same place at the distal end of ileum and fixed in 4% formaldehyde. After embedding in paraffin, 4 *μ*m sections were stained with hematoxylin and eosin before assessment by light microscopy (original magnification ×200, Olympus BX50; Olympus Optical, Tokyo, Japan).

Using the improved Chiu score method [21] to evaluate intestinal mucosal damage, higher scores are interpreted to indicate more severe damage. Criteria of Chiu grading system consist of 5 subdivisions according to the changes of villus and gland of intestinal mucosa: grade 0, normal mucosa; grade 1, development of subepithelial Gruenhagen's space at the tip of villus; grade 2, extension of the space with moderate epithelial lifting; grade 3, massive epithelial lifting with a few denuded villi; grade 4, denuded villi with exposed capillaries; and grade 5, disintegration of the lamina propria, ulceration, and hemorrhage.

### 2.6. Assessment of Pulmonary Edema

The left lung was harvested. After the lung wet weight was measured, the lungs were placed in a calorstat at 60°C for 48 h, and then the specimen was reweighed. The pulmonary edema was estimated by lung wet/dry weight ratio [22].

### 2.7. Immunohistochemical Assessment

Paraffin-embedded lung sections were stained using the streptavidin-biotin complex immunohistochemistry technique for HO-1 and Nrf2 detection. Brown staining in the cytoplasm and/or nucleus was considered an indicator of positive expression. With the Image-Pro Plus version 6.0, results were evaluated semiquantitatively according to optical density values of positive expression.

### 2.8. Western Blot Analysis

The right lungs were removed and nuclear fractions were prepared using a Nuclear and Cytoplasmic Protein Extraction Kit (Beyotime Institute of Biotechnology, China) according to the manufacturer's protocol. Western blot analysis was carried out as described [23]. Primary antibodies (working concentration) used were rabbit polyclonal antibodies against mice Nrf2 (1 : 2000, H-300, Santa Cruz Biotechnology, CA), HO-1 (1 : 1000, H-105, Santa Cruz Biotechnology, CA), and Lamin B1 (1 : 2000, H-90, Santa Cruz Biotechnology, CA). The HRP-conjugated secondary antibody was goat anti-rabbit IgG (Beyotime Institute of Biotechnology, China) used at 1 : 2000. Lamin B was used as an internal control. The ECL Western blotting detection reagents (Beyotime Institute of Biotechnology, China) were used for visualization of the protein bands. The intensities of the bands were analyzed with quantity one software (Bio-Rad, Hercules, CA).

### 2.9. Determination of Tissue Tumor Necrosis Factor Alpha (TNF-*α*), Interlukin-6 (IL-10), and Interlukin-6 (IL-6)

Tissue levels of TNF-*α*, IL-10, and IL-6 were determined using commercially available ELISA kits (R&D, Minneapolis, MN) according to manufacturer's instructions. The results were expressed as pg/mL.

### 2.10. Determination of Tissue MDA Level and SOD Activity

The right lung tissues were homogenized on ice in normal saline. The homogenates were centrifuged at 4000 g·min^−1^ at 4°C for 10 min. The MDA level in the supernatants was determined by the measurement of thiobarbituric acid-reactive substances levels (assay kits were supplied by Nanjing Jiancheng Corp., China) as previously described [24]. The results were calculated as nmol·100 mg^−1^ protein. The SOD activity in the supernatants was evaluated by inhibition of nitroblue tetrazolium (NBT) reduction by O^2−^ generated by the xanthine/xanthine oxidase system (assay kits were supplied by Nanjing Jiancheng Corp., China) in accordance with the previous method [24]. The results were expressed by U·100 mg^−1^ protein.

### 2.11. Statistical Analysis

Data are presented as mean ± SD. Statistical comparison among multiple groups was performed by one-way analysis of variance (ANOVA) followed by Newman-Keuls multiple comparison test using the GraphPad Prism 5.0 software (GraphPad Software Inc., San Diego, CA, USA). A value of *P* < 0.05 was considered statistically significant.

## 3. Results

### 3.1. Histopathological Assessment of Intestines

**Figure 2:**
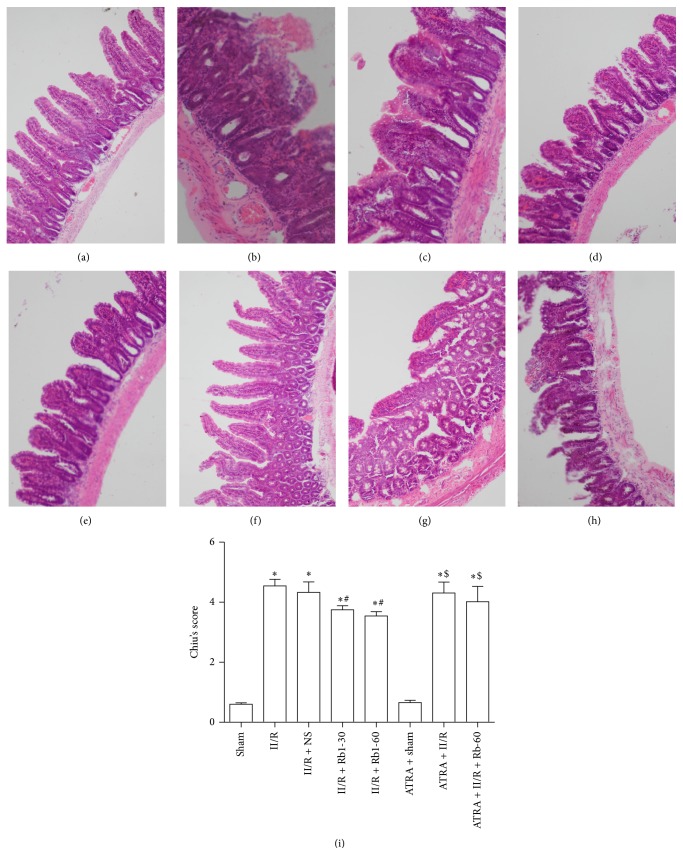
Intestinal histologic evaluation in the groups. ((a)–(h)) Histopathologic changes of the small-intestinal mucosa were observed under light microscopy (hematoxylin and eosin, ×200). (a) Sham group, (b) II/R group, (c) II/R + NS group, (d) II/R + Rb1-30 group, (e) II/R + Rb1-60 group, (f) ATRA + Sham group, (g) ATRA + II/R group, and (h) ATRA + II/R + Rb1-60 group. Data are mean ± SD, *n* = 10; ^*^ *P* < 0.05 versus Sham group, ^#^ *P* < 0.05 versus II/R group, and ^$^ *P* < 0.05 versus II/R + Rb1-60 group.

The II/R group showed edema in the villi, inflammatory cells infiltration, and damaged areas interspersed with hemorrhage. In addition, the gap between epithelial cells significantly increased and capillaries and lymph vessels were markedly dilated. Normal villi were seen in the intestine of the Sham group and ATRA + Sham group under the light microscope. Ginsenoside Rb1 at the dose of 30 mg/kg and 60 mg/kg both significantly attenuated the histological intestine injury (Figure 2). However, there is little amelioration of the intestine injury induced by II/R in the ATRA + II/R + Rb1-60 group.

### 3.2. Pathologic Alternations of Lung Tissue

**Figure 3:**
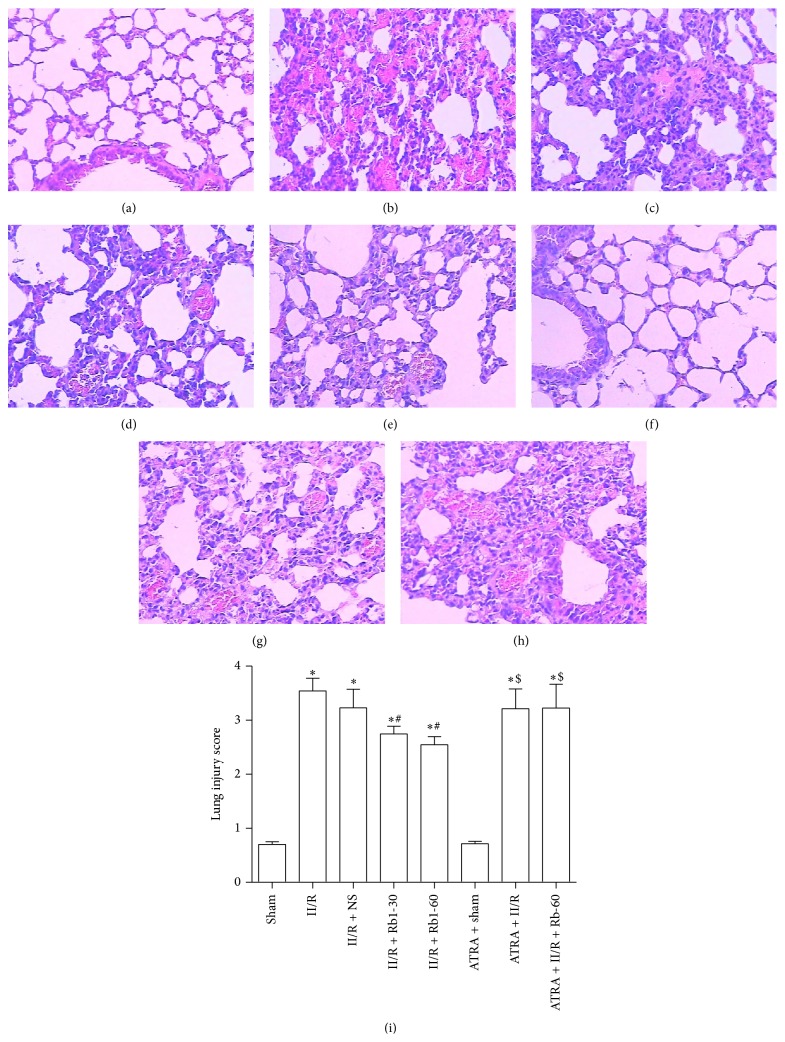
Histopathologic changes in mice lung under light microscopy (hematoxylin and eosin, ×200). (a) Sham group, (b) II/R group, (c) II/R + NS group, (d) II/R + Rb1-30 group, (e) II/R + Rb1-60 group, (f) ATRA + Sham group, (g) ATRA + II/R group, and (h) ATRA + II/R + Rb1-60 group. Data are mean ± SD, *n* = 10; ^*^ *P* < 0.05 versus Sham group, ^#^ *P* < 0.05 versus II/R group, and ^$^ *P* < 0.05 versus II/R + Rb1-60 group.

The lungs from II/R group showed damaged areas interspersed with hemorrhage, inflammatory cell infiltration, and pulmonary edema, while little damage was seen in the lungs of the sham group and ATRA + Sham group under the light microscope. Ginsenoside Rb1 at the both doses of 30 mg/kg and 60 mg/kg significantly attenuated the histological lung injury (Figure 3). However, there is little amelioration of the lung injury induced by II/R in the ATRA + II/R + Rb1-60 group. This indicates that ATRA attenuated the protective action of ginsenoside Rb1 against II/R induced lung damage in the mice.

### 3.3. Changes of Lung Wet/Dry Weight Ratio

**Figure 4:**
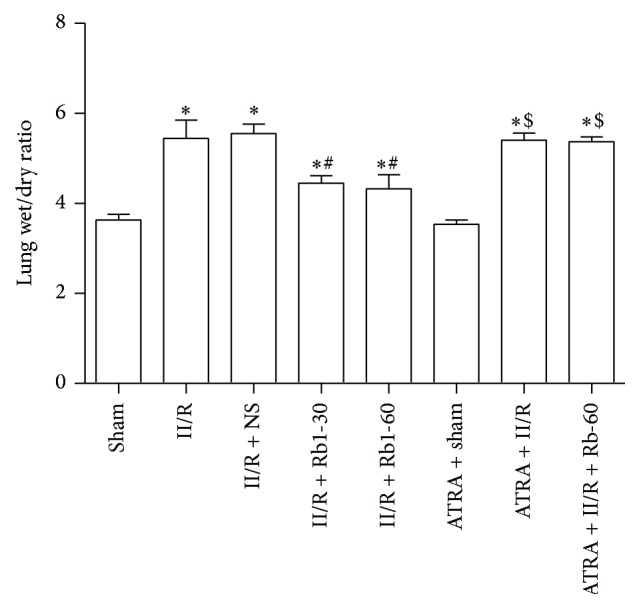
The effects of ginsenoside Rb1 on the lung wet/dry weight ratio. Data are mean ± SD, *n* = 5; ^*^ *P* < 0.05 versus Sham group, ^#^ *P* < 0.05 versus II/R group, and ^$^ *P* < 0.05 versus II/R + Rb1-60 group.

We next assessed the lung wet/dry weight ratio as indicators of lung permeability damage. As shown in Figure 4, the lung wet/dry weight ratio was significantly higher in the II/R group than the Sham group (*P* < 0.05). Compared with the II/R group, the lung wet/dry ratio was decreased significantly after the treatment of ginsenoside Rb1 (*P* < 0.05, II/R + Rb1-30 or II/R + Rb1-60 versus II/R). This decrease was reversed by administration of ATRA (*P* > 0.05, ATRA + II/R + Rb1-60 versus II/R). There was no significant difference between the Sham and ATRA + Sham group or II/R and II/R + NS group (*P* > 0.05).

### 3.4. Changes of the Level of MDA and the Activity of SOD

**Figure 5:**
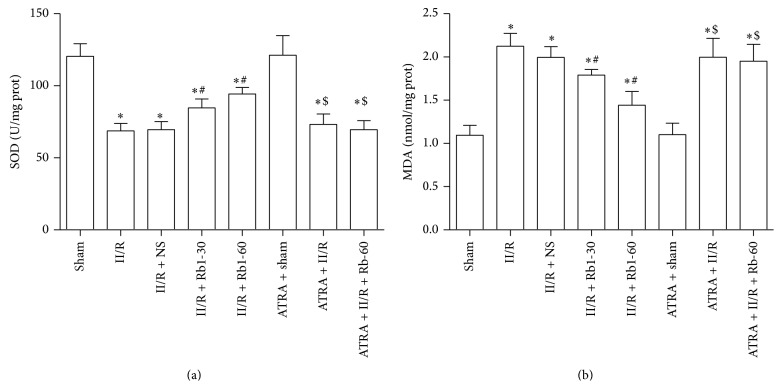
The changes of lung tissue SOD activity and MDA levels. Data are mean ± SD, *n* = 10; ^*^ *P* < 0.05 versus Sham group, ^#^ *P* < 0.05 versus II/R group, and ^$^ *P* < 0.05 versus II/R + Rb1-60 group.

Oxidative stress has been proposed as an important mechanism of the development of II/R induced organ damage. Reperfusion or reoxygenation will activate recovery of aerobic metabolism and results in an overload of reactive oxygen species (ROS). Robust ROS generation generates excessive hydroxyl radicals, which are very unstable and have a high potential to damage cellular structures, enzymes, or channel proteins on the cellular membrane. We examined the effects of ginsenoside Rb1 on lung tissue lipidic peroxidation product (MDA) levels and the antioxidative SOD activity. As shown in Figure 5, treatment with 30 mg/kg and 60 mg/kg ginsenoside Rb1 significantly reduced MDA levels and increased SOD activity, and this effect was inhibited by administration of ATRA.

### 3.5. Changes of Tissue TNF-*α*, IL-10, and IL-6 Levels

**Figure 6:**
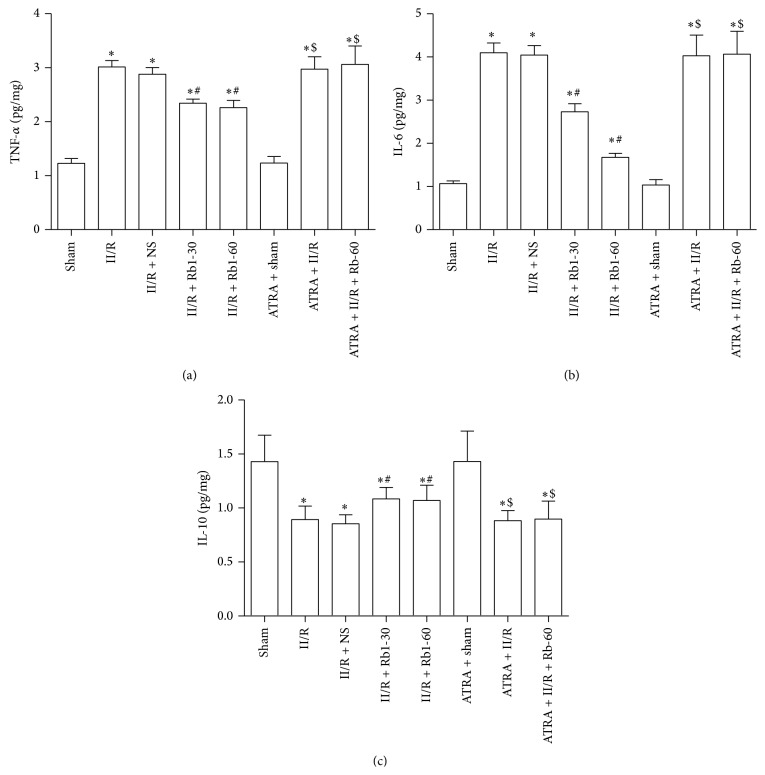
Cytokine levels in lung from mice. Cytokine levels were determined in lung homogenate using multiplex analysis: (a) TNF-*α*, (b) IL-6, and (c) IL-10. Data are mean ± SD, *n* = 10; ^*^ *P* < 0.05 versus Sham group, ^#^ *P* < 0.05 versus II/R group, and ^$^ *P* < 0.05 versus II/R + Rb1-60 group.

In a number of clinical studies, microinflammation has been found to be associated with processes that may be related to II/R caused injury. As shown in Figure 6, the level of tissues TNF-*α* and IL-6 in the II/R group was significantly higher than that in the Sham group. However, the level of tissue IL-10 was significantly reduced in the II/R group compared to that in the Sham group. Treatment with 30 mg/kg and 60 mg/kg ginsenoside Rb1 significantly reduced TNF-*α* and IL-6 levels and increased IL-10 levels. After treatment with ATRA, this effect was inhibited.

### 3.6. Effects of Ginsenoside Rb1 on HO-1 and Nrf2 Expression in Lung Tissue by Immunohistochemical Detection

**Figure 7:**
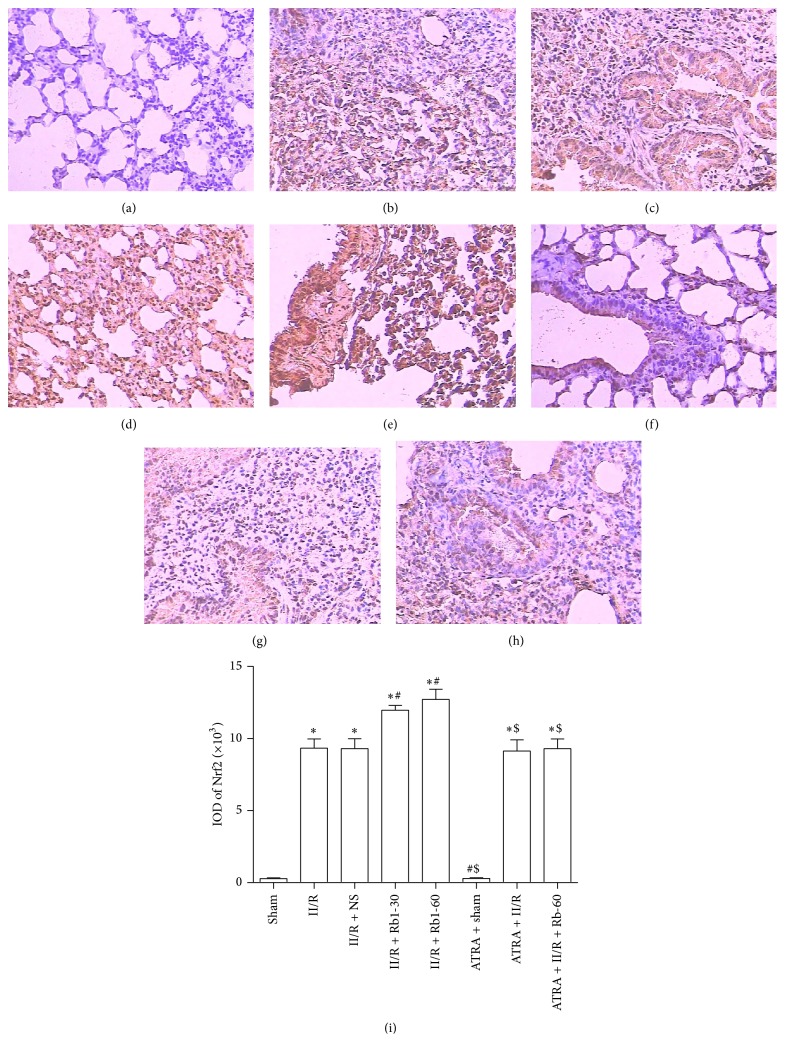
Expression of Nrf2 in the lung tissue under the light microscope (streptavidin-biotin complex immunohistochemistry, ×200). (a) Sham group, (b) II/R group, (c) II/R + NS group, (d) II/R + Rb1-30 group, (e) II/R + Rb1-60 group, (f) ATRA + Sham group, (g) ATRA + II/R group, and (h) ATRA + II/R + Rb1-60 group. Data are mean ± SD, *n* = 5; ^*^ *P* < 0.05 versus Sham group, ^#^ *P* < 0.05 versus II/R group, and ^$^ *P* < 0.05 versus II/R + Rb1-60 group.

**Figure 8:**
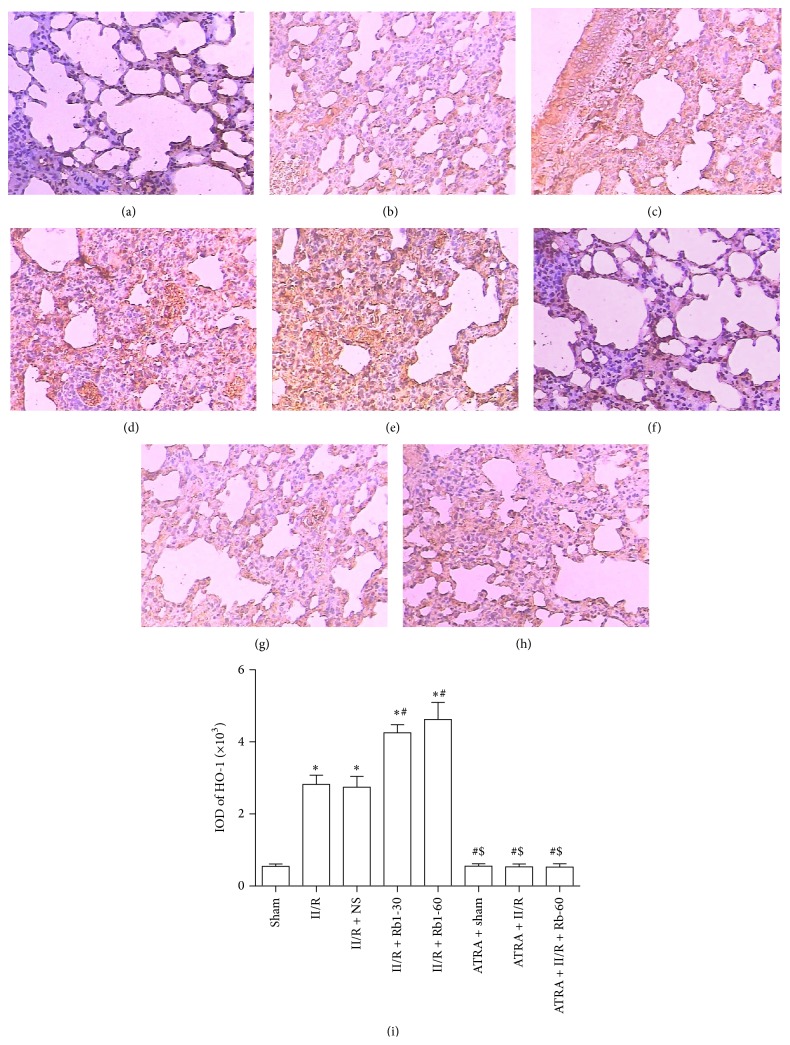
Expression of HO-1 in the lung tissue under the light microscope (streptavidin-biotin complex immunohistochemistry, ×200). (a) Sham group, (b) II/R group, (c) II/R + NS group, (d) II/R + Rb1-30 group, (e) II/R + Rb1-60 group, (F) ATRA + Sham group, (g) ATRA + II/R group, and (h) ATRA + II/R + Rb1-60 group. Data are mean ± SD, *n* = 5; ^*^ *P* < 0.05 versus Sham group, ^#^ *P* < 0.05 versus II/R group, and ^$^ *P* < 0.05 versus II/R + Rb1-60 group.

The lung tissue was obtained to measure the expression of Nrf2 and HO-1 by immunohistochemical assay. In the II/R group, both cytoplasm and nuclei of the lung tissue showed Nrf2 expression, but the expression of HO-1 was showed in the cytoplasm. Compared with the Sham group, the expression of Nrf2 and HO-1 in the II/R group increased significantly. After treatment with ginsenoside Rb1 at dose 30 or 60 mg/kg, the expression of Nrf2 and HO-1 was much higher than that in the II/R group. In the ATRA + II/R and ATRA + II/R + Rb1-60 groups, Nrf2 was also expressed obviously in the cytoplasm and nuclei, though mild expression of HO-1 could be seen in the cytoplasm of lung tissue in these groups (Figures 7 and 8).

### 3.7. Effects of Ginsenoside Rb1 on Cytoplasmic HO-1 and Nuclear Nrf2 Expression in Lung Tissue by Western Blotting Analysis

**Figure 9:**
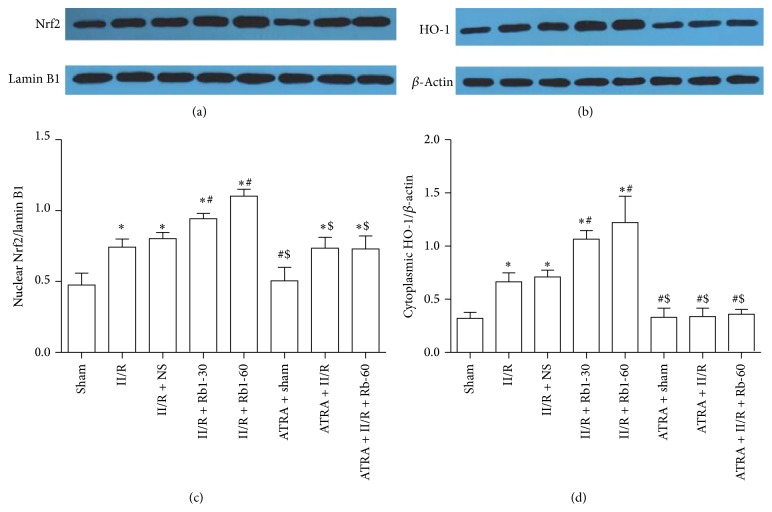
Western blotting analysis of the presence of Nrf2 in nuclear proteins and HO-1 in cytoplasmic proteins in the mice lung tissue. Data obtained from quantitative densitometry were presented as mean ± SD. *n* = 10, ^*^ *P* < 0.05 versus Sham group, ^#^ *P* < 0.05 versus II/R group, and ^$^ *P* < 0.05 versus II/R + Rb1-60 group.

To further confirm the protective effect of ginsenoside Rb1 on the lung tissue against II/R injury, protein expression of nuclear Nrf2 and cytoplasmic HO-1 was examined by Western blot. As shown in Figure 9, Nrf2 and HO-1 expression were both increased markedly in the II/R group as compared with the Sham group. II/R with Rb1 intervention further increased the expression of Nrf2 and HO-1 significantly. ATRA administration has no effects on the cytoplasmic HO-1 expression as compared with the Sham group. This indicated that Rb1 induced cytoplasmic HO-1 expression was inhibited by ATRA. There was no significant difference in Nrf2 expression between the ATRA + II/R group and the II/R group or between the ATRA + II/R + Rb1-60 group and the II/R group.

## 4. Discussion

In this study, we have demonstrated in a mice model that 45 min occlusion of SMA followed by 2 h of reperfusion caused significant lung injury as evidenced by pathologic morphologic changes seen in the lung tissue, as well as the increased lung wet/dry ratio, which is in accordance with the previous reports [25]. We found that postischemia treatment with ginsenoside Rb1 enhanced Nrf2 translocation to the nucleus in the lung tissues of mice and Rb1 treatment could reduce pulmonary morphologic damage, alleviate injuries induced by oxidative stress, and modulate inflammatory reactions. Further, Nrf2 function inhibition with ATRA reverted the pulmonary protective effects of ginsenoside Rb1, indicating that ginsenoside Rb1 confers its respiratory protection via Nrf2/ARE signaling in the II/R induced acute lung injury.

The mechanisms of acute lung injury induced by II/R are complex. It is thought that the damage of intestinal mucosal barrier following II/R causes the dislocation of bacteria or endogenous endotoxin, thus leading to increased oxidative stress and systemic inflammatory reaction, which is one of the main reasons for acute lung injury.

Ginseng is one of the most widely used herbal medicines. Ginsenosides, the major active ingredient of ginseng, have been noticed for their multiple pharmacological effects on antioxidation, signal transduction pathways, and interaction with receptors [26]. Oxidative stress refers to the mismatched redox equilibrium between the production of free radicals and the ability of cells to defend against them. One feasible way to prevent free radical mediated cellular injuries is to augment the oxidative defense capacity through intake of antioxidants. Moreover, the induction of endogenous phase II detoxifying enzymes or antioxidative proteins seems to be a reasonable strategy for delaying disease progression. Activation of Nrf2/ARE plays an important role in protecting cells from oxidative stress [27, 28]. The ability of Nrf2 to upregulate the expression of antioxidant genes via ARE suggests that increasing Nrf2 activity may provide a useful system for combating oxidative insults. Several recent reports have demonstrated that coordinate upregulation of ARE-driven genes protects organs from ischemia reperfusion injury [29–31]. Accumulating evidence also suggests that upregulation of HO-1 expression and the subsequent increase in HO activity may confer an adaptive survival response against oxidative insults. Our previous studies showed that Rb1 reduces renal apoptosis and alleviates renal dysfunction after II/R in part through the Nrf2/ARE pathway [32]. Wang et al. demonstrated that Rb1 attenuates lung injury induced by II/R via inhibiting NF-*κ*B activation [33]. To determine the mechanism by which postischemia treatment with Rb1 reduces II/R-induced ROS generation, we examined the effect of ginsenoside Rb1 on Nrf2 and HO-1 expression in mice lung tissues. Our present study demonstrated that Rb1 increased nuclear Nrf2 protein and cytoplasmic HO-1 protein expressions in lung tissues of mice after II/R. The increase of HO-1 expression by Rb1 conferred cytoprotection against II/R induced oxidative stress. In addition, previous studies have shown that ATRA does not affect the half-life of Nrf2 or its nuclear translocation. ATRA inhibits Nrf2 function by stimulating the formation of Nrf2:RAR*α*-containing complexes that do not bind to the ARE [18]. We showed that ATRA, as a potent inhibitor for combination of Nrf2 with ARE, partially reversed the protective effects of Rb1, thus providing further evidence for Nrf2/ARE as a possible cytoprotective pathway for Rb1.

Ginseng extract was reported to have immunomodulatory effects [34]. Smolinski and Pestka [35] reported that immunologic effects include modulation of lipopolysaccharide-induced proinflammatory cytokine production in vitro and in vivo by ginsenoside Rb1. This was also confirmed with our study in which Rb1 significantly reduced the tissue level of TNF-*α*, IL-6, and IL-10. These results show that Rb1 may have multiple mechanisms of action that affect cytoprotection by both reducing ROS generation and increasing the anti-inflammatory effect.

In summary, our present study indicates that ginsenoside Rb1 alleviates acute lung injury following II/R via activating Nrf2/ARE pathway. The experiment data may help us further understand the pharmacological effects of Rb1 and also suggest a new therapeutic target to protect the body from II/R injury. However, further studies need to be performed in transfection of lung endothelial cells and intestinal epithelial cells with the siRNA and expressing plasmid for Nrf2 to confirm the findings of the current study.
