# Correction to “Ginsenoside Rb1 Treatment Attenuates Pulmonary Inflammatory Cytokine Release and Tissue Injury Following Intestinal Ischemia Reperfusion Injury in Mice”

**DOI:** 10.1155/omcl/9782363

**Published:** 2026-07-29

Y. Jiang, Z. Zhou, Q. Meng, et al., “Ginsenoside Rb1 Treatment Attenuates Pulmonary Inflammatory Cytokine Release and Tissue Injury Following Intestinal Ischemia Reperfusion Injury in Mice,” *Oxidative Medicine and Cellular Longevity*, 2015, 843721, https://doi.org/10.1155/2015/843721.

In the article, it was reported on PubPeer [1] that Figure 2a appears to be a zoomed‐out image taken from Figure 1a of a previous publication of many of the authors [2], cited in the article as reference 32. The authors wish to clarify that both studies were conducted concurrently as part of a larger research program, and that Sham control animals were shared between the two studies. A revised Figure 2 legend is as follows, which acknowledges the above.

Intestinal histologic evaluation in the groups. ((a)–(h)) Histopathologic changes of the small‐intestinal mucosa were observed under light microscopy (hematoxylin and eosin, ×200). (a) Sham group. The representative image was previously published in Ref. [32]. The two studies were conducted concurrently as part of a larger research program and shared the same Sham control animals, with each study focusing on a different target organ (kidney and lung, respectively). (b) II/R group, (c) II/R + NS group, (d) II/R + Rb1‐30 group, (e) II/R + Rb1‐60 group, (f) ATRA + Sham group, (g) ATRA + II/R group, and (h) ATRA + II/R + Rb1‐60 group. Data are mean ± SD, *n* = 10;  ^∗^ *p* < 0.05 versus Sham group, ^#^ *p* < 0.05 versus II/R group, and ^$^ *p* < 0.05 versus II/R + Rb1‐60 group.

We apologize for this error.
